# Construction and In Vitro Evaluation of a Tumor Acidic pH-Targeting Drug Delivery System Based on *Escherichia coli* Nissle 1917 Bacterial Ghosts

**DOI:** 10.3390/bioengineering9090433

**Published:** 2022-09-02

**Authors:** Yi Ma, Qiying Liu, Aihua Hu, Shoujin Jiang, Sijia Wang, Ran Liu, Kun Han, Jufang Wang

**Affiliations:** 1 School of Biology and Biological Engineering, South China University of Technology, Guangzhou 510006, China; 2 Guangdong Provincial Key Laboratory of Fermentation and Enzyme Engineering, South China University of Technology, Guangzhou 510006, China; 3 The Third Affiliated Hospital of Guangzhou Medical University, Guangzhou 510140, China; 4 Jiangsu Key Biotechnology Co., Ltd., Xuzhou 221100, China

**Keywords:** bacterial ghosts, drug delivery system, phage α3, Lpp-OmpA displays system, ATRAM

## Abstract

Synthetic nanocarriers are a promising therapeutic delivery strategy. However, these systems are often hampered by inherent disadvantages such as strong biotoxicity and poor biocompatibility. To overcome these issues, biological carriers with commonly used chemotherapy drugs have been developed. In this work, engineered bacterial ghosts (BGs) originated from probiotic *Escherichia coli* Nissle 1917 (EcN) were devised to specifically target acidic extracellular environments of tumor tissue. To improve the production efficiency and safety, a novel lysis protein E from phage α3 was applied to produce EcN BGs under high growth densities in high quality. In addition, the acidity-triggered rational membrane (ATRAM) peptides were displayed in EcN BGs to facilitate specific cancer cell internalization within the acidic tumor microenvironment before drug release. In conclusion, the engineered EcN BGs offer a promising means for bionic bacteria construction for hepatocellular carcinoma therapy.

## 1. Introduction

Cancer is a major global public health problem. According to the Global Cancer Statistics 2022 report, the novel coronavirus 2019 (COVID-19) pandemic has delayed cancer diagnoses and treatments, and finally led to an increase in mortality [1]. Hepatocellular carcinoma is one of the commonest among the various types of cancer and the leading cause of cancer death in China and the USA [2]. Doxorubicin (DOX) has proven to be one of the most effective treatments against various cancers including hepatocellular carcinoma [3]. The antitumor mechanisms of DOX include inhibition of a topoisomerase related to DNA synthesis and induction of DNA breaks. However, its non-specific targeting of various cells leads to the occurrence of many adverse reactions such as cardiotoxicity [4], myelosuppression, and immunosuppression, limiting its clinical application [5].

It is well-known that the normal physiological pH of tissues in the human body is close to 7.4, but tumor tissues are weakly acidic due to the proliferation of tumor cells (pH 6.5–7.0) [6]. Thus, synthetic nanocarriers (e.g., liposomes, micelles, polymersomes, nanoparticles) targeted at this tumor acidic microenvironment have been increasingly investigated for the treatment of cancers [7]. However, few such nano-based cancer drugs have been successfully developed for use in the clinic, and their efficacy is often hampered by several biological barriers and large-scale production difficulties [8]. Biological carriers derived from diverse natural sources such as bacteria [9,10] and various cells [11,12] within the body have attracted attention. Bacteria are the most abundant organisms on earth, and the use of tumor-targeting bacteria can be traced back to the pioneering work of William Coley, who discovered tumor regression in some cancer patients who had received bacterial injections [13]. Ever since that discovery, researchers have attempted to leverage bacteria for medical advancements, as they are biodegradable and have evolved specific functions in vivo that are desirable for drug delivery systems such as immune system evasion and selective colonization [14]. However, colonization location uncertainty and the possibility of mutations occurring in live bacteria may lead to uncertain side effects in the host [15].

Various methods have been used to improve the safety of bacterial treatment carriers such as the development of auxotrophic mutations and bacterial safety agents [16,17,18] or the employment of non-pathogenic bacterial strains (such as EcN) [19]. Therefore, bacterial derivatives such as BGs [20], which maintain bacteria-like tumor-targeting properties and immune regulating functions, but avoid infectious threats, have been suggested as vectors to improve systemic safety in antitumor therapeutics.

BGs are nonliving empty cell envelopes formed by the expression of the lysis E gene of bacteriophage φX174 [21]. The entire cytoplasmic contents of bacteria including their genetic material are expelled from the membrane channel owing to the change in osmotic pressures, which means BGs pose no horizontal gene transfer danger [22]. Moreover, the complete natural surface structures of BGs, including fimbriae and outer membrane proteins, resemble those in their living counterparts [23] and endow these carriers with different properties from those in synthetic delivery carriers [24]. The empty inner space of BGs can be filled with drugs [25], proteins [26], DNA [27], or other substances, providing a wide application prospect in biomedicine. Paukner et al. showed that the *Mannheimia haemolytica* ghosts carried moderate hydrophilic doxorubicin (DOX) with a stronger anti-proliferation activity against Caco-2 cells than free DOX [28]. Similar results were found in *E. coli* BL21 ghosts loaded with 5-fluorouracil [29], which effectively targeted Caco-2 cells with promising cytotoxic activity. Further, Zhu et al. revealed that the releases of cytochrome C and active caspase-3 caused by epothilone B-bacterial ghosts were higher than those caused by the free epothilone B and conferred anti-proliferative properties to the epothilone B-bacterial ghosts that were mediated by mitochondria apoptosis pathways [25].

Considering the heterogeneity of tumors, seeking therapeutic cargos targeted to acidic tissues might be of general use for fighting cancer. Peptides are attractive molecules for the delivery of cargo to acidic diseased tissues [30,31]. Thus, the pH-sensitive acidity-triggered rational membrane (ATRAM) peptide, which is improved on the basis of a low-pH insertion peptide (pHLIP), has been designed [30]. The modification allows ATRAM to achieve higher solubility at high concentrations (>200 µM) and enhances the membrane targeting of ATRAM under acidic conditions [32]. It is a specific therapeutic agent for targeting acidic tumor microenvironments.

**Scheme 1:**
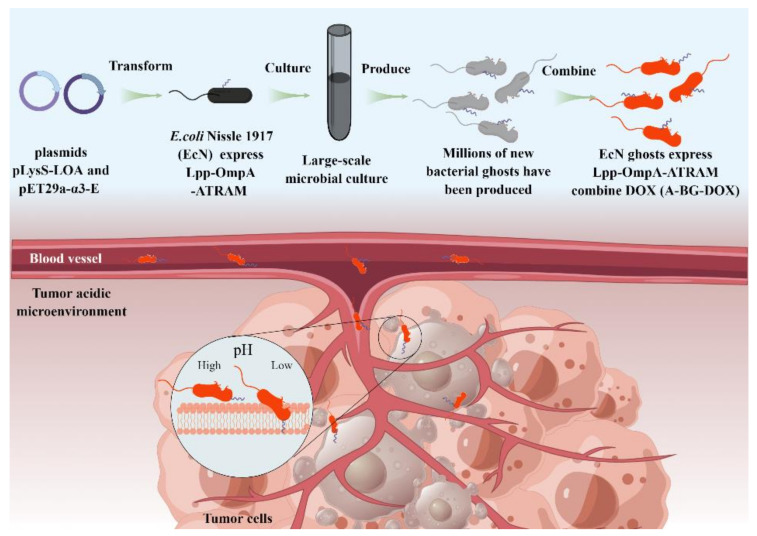
Illustration of large-scale production of and tumor targeting by A-BG-DOX, a pH-responsive bacterial ghost for killing tumor cells.

In this study, we demonstrated the simple production process of engineered BGs, and a novel bacterial ghost-based delivery system capable of targeting acidic tumor microenvironments via the ATRAM peptide (Scheme 1). And we offer a promising means for bionic bacteria construction for hepatocellular carcinoma therapy.

## 2. Materials and Methods

### 2.1. Plasmids, Bacterial Strains, and Cell Culture

Plasmid pET29a-α3-E, derived from phage α3 (synthesized by Azenta; Suzhou, China), was transformed into *E. coli* BL21(DE3) and EcN/Δ*tnaA*::T7 RNAP to express bacterial lysis protein. Plasmid pET29a-φX174-E, derived from phage φX174, was developed in our lab. The pLysS plasmid vector containing the nucleotide sequence of Lpp-OmpA-ATRAM (pLysS-LOA) was also synthesized by Azenta (Suzhou, China). The plasmids from this study were contributed to Addgene (www.addgene.org) with promotion numbers recorded in the Table S1, accessed on 20 June 2022.

*E. coli* BL21(DE3), the general host for our lysis protein E experiments, was purchased from Tiangen Biotech (Beijing, China). EcN/Δ*tnaA*::T7 RNAP was developed in our lab. Luria-Bertani (LB) medium for bacteria cultivation was used and supplemented with antibiotics as required. All bacterial strains were stored at −80 °C in LB with 30% glycerol.

The human hepatoma HepG2 cell line was sourced from our lab and grown in dulbecco’s modified eagle medium (DMEM) medium (Gibco, Grand Island, NY, USA) supplemented with 10% fetal bovine serum (FBS; Lonsera, Ciudad de la Costa, Uruguay) and 1% penicillin/streptomycin (Gibco, USA). The human hepatocyte L02 cell line was sourced from our lab and grown in roswell park memorial institute (RPMI) 1640 medium (Gibco, USA) supplemented with 10% FBS and 1% penicillin/streptomycin. The cells were grown in a humidified incubator with 5% CO_2_ at 37 °C.

### 2.2. Effect of Lysis Protein E in Bacteria

*E. coli* BL21(DE3) is an efficient host strain to express foreign proteins with a clear genetic background and is suitable for our experiments with lysis protein E. For this study, we transformed the plasmids pET29a-α3-E and pET29a-φX174-E into *E. coli* BL21(DE3) cells and incubated the bacteria in LB broth containing kanamycin (50 µg/mL). When the recombinant strains in culture achieved the required OD_600_ values, we added 1 mM IPTG to induce the expression of lysis protein E. We ran an agarose gel electrophoresis to evaluate the genome in the bacteria. The DNA was loaded into a 1% agarose gel (Invitrogen, Carlsbad, CA, USA) and run at 120 V for 20 min using a power supplier unit (Bio-Rad, Hercules, CA, USA). The gel was analyzed using a gel documentation system.

### 2.3. Western Blot of Lysis Protein E

We carried out western blot to verify the expression of the lysis protein E. Briefly, the overnight cultured *E. coli* BL21(DE3) cells were transferred to fresh LB medium at a 1% ratio. We added 1 mM IPTG to induce the protein E during the logarithmic growth period and we continued the cultures for 15, 30, and 60 min. Next, we collected the samples by centrifugation at 12,000 rpm for 2 min and resuspended the cell pellets in lysis buffer. The cell mixture was lysed in a sonicator for 5 min and then boiled for 10 min. Then, we loaded 10 µL protein samples on a 16% Tricine gel for SDS-PAGE and transferred the separated proteins onto a nitrocellulose membrane (GE Healthcare Bio-Sciences, Piscataway, NJ, USA) in a Bio-Rad Transblot Turbo system at 80 V for 1 h. After incubation with the 5% (*w*/*v*) non-fat milk powder in TBST buffer (Sangon Biotech, Shanghai, China) for 1 h at room temperature, the membrane was washed twice with TBST buffer for 15 min each time. Next, we incubated the membrane with anti-Strep-Tag II monoclonal antibody (Abbkine, Wuhan, China) at 4 °C overnight, and the next day, with HRP-conjugated goat anti-mouse IgG H&L (Abcam, Cambridge, UK) for 1 h at room temperature. Finally, the Super Signal West Pico Chemiluminescent Substrate (Thermo Fisher Scientific, Waltham, MA, USA) was used to visualize the immunoreactive protein.

### 2.4. Identification of the Protein Display System of the Engineered EcN Using SDS-PAGE and Immunofluorescence

The plasmids pET29a-α3-E and pLysS-LOA were transformed into EcN/Δ*tnaA*::T7 RNAP cells by electroporation (1.5 kV, 200 Ω, 25 µF) and the bacteria were incubated in LB broth containing kanamycin (50 µg/mL) and chloramphenicol (25 µg/mL). EcN/Δ*tnaA*::T7 RNAP bearing no pLysS-LOA plasmid was used as control. The overnight cultures of EcN/Δ*tnaA*::T7 RNAP were transferred to fresh LB media culture tubes. We assessed bacterial growth and lysis conditions by measuring the OD_600_, and we collected samples every hour during the process. Next, we performed SDS-PAGE to assess protein samples. Briefly, the bacteria were harvested and lysed by ultrasonication, and we loaded the samples on a 12% SDS-PAGE gel for electrophoresis.

We conducted immunofluorescence experiments to identify ATRAM on the cell membranes of the EcN/Δ*tnaA*::T7 RNAP. A 1 mL sample of the exponential growth of the bacteria strain was collected by centrifugation at 4000 rpm for 5 min and washed three times using PBS to remove the remaining LB medium. Then, after being blocked with 200 µL blocking buffer (BSA) (Sangon Biotech, China) for 1 h at room temperature, the bacteria were collected at 4000 rpm for 5 min, washed three times using PBS and incubated with mouse monoclonal ANTI-FLAGM2 antibody (Sigma, St. Louis, MO, USA) (dilution 1:3000) at 4 °C overnight. Next, the bacteria were incubated with goat anti-mouse IgG H&L (Alexa Fluor 488) (Abcam, UK) at a dilution of 1:500 for 2 h at room temperature. The immunofluorescence on the membrane surfaces of the EcN/Δ*tnaA*::T7 RNAP was captured using a confocal laser scanning microscope Leica TCS SP8 (Leica, Wetzlar, Germany).

### 2.5. Scanning Electron Microscopy (SEM) Analysis

Scanning electron microscopy was used to observe the transmembrane channels and morphology of BGs and was performed as previously described [33]. After IPTG induction for 4 h, the EcN/Δ*tnaA*::T7 RNAP/pLysS-LOA and its ghosts (A-BGs) were collected and centrifuged at 4000 rpm for 15 min to remove the LB medium. The samples were resuspended in 1 mL 2.5% glutaraldehyde special electron microscopic fixative (Phygene, Fuzhou, China) and stored at 4 °C overnight as a prefixation step. Then, the cells were washed three times with deionized water, dehydrated three times with a graded series (70%, 85%, 95%) of ethanol and finally soaked in 100% ethanol. Following the final dehydration, the samples were dried in an Autosamdri-815 critical point drier and then coated with a gold sputter coater. We used Merlin scanning electron microscopy (Zeiss, Jena, Germany) to observe the morphology of the bacterial cells and BGs.

### 2.6. Transmission Electron Microscopy (TEM) Analysis

We used transmission electron microscopy to observe the outflow of bacterial contents and the morphology of the A-BGs. First, 10 µL of the prepared samples were adsorbed for 5 min to a prepared carbon film copper mesh. Next, we added 10 µL of 3% phosphotungstic acid stain solution and waited for 5 min. The unbound dye solution was absorbed using a pipette and softly washed two times with ultrapure water. Finally, the TEM images were acquired under a Talos L120C transmission electron microscope (Thermo Fisher Scientific, USA).

### 2.7. Drug Packaging

To evaluate drug packaging into the A-BGs, we loaded the lyophilized A-BGs with DOX using a modified published protocol [34]. We collected the A-BGs by centrifugation at 4000 rpm for 15 min and washed them three times with PBS. The obtained ghosts were lyophilized with mannitol and stored at −20 °C until use. Approximately 30 mg of the lyophilized A-BGs were incubated with 0, 2.5, 5, or 7.5 mg/mL of DOX (Meilunbio, China) and 0.5 M Tris-HCL buffer (pH 8.0), or alternatively with 5 mg/mL of DOX and 0.5 M Tris-HCL buffer at different pH values (6.8 and 7.4), and all the samples were left to stand for 1 h at 37 °C. Moreover, lyophilized A-BGs were also incubated with 5 mg/mL DOX and 0.5 M Tris-HCL buffer (pH 8.0) and then left to stand for 15, 30, or 60 min at 37 °C, or for 60 min at 4, 16, 25, 37, and 55 °C. Subsequently, we washed the A-BGs with Tris-HCl buffer three times and finally with ultrapure water before lyophilizing them again. The packed A-BGs were suspended in 500 μL 10% SDS solution, before incubating them for 10 min at 65 °C. We added 500 µL of diluted sulfuric acid to the samples and further incubated them at 65 °C for 10 min. The DOX contents of the loaded A-BGs (A-BG-DOX) were determined by ultraviolet spectrophotometry. The loading capacity and encapsulation efficiency were calculated by Equations (1) and (2):

Loading capacity (%) = 100% × W_DOX_/W_BGs-DOX_ (1)

Encapsulation Efficiency (%) = 100% × W_DOX_/W_DOX added_ (2)

### 2.8. Drug Release Profile of BGs

We used a dynamic dialysis method to assess the in vitro DOX release from the A-BGs. We poured a 1 mL suspension of A-BG-DOX into a dialysis bag. The dialysis sack was closed on both ends. We dipped the dialysis bag in 19 mL PBS (pH 5.5, 6.5, 7.4) maintained at 37 °C in a shaker (150 rpm) for 72 h. To keep the dialysis system functioning, we replaced every 1 mL sample taken from the dialysate with 1 mL of fresh medium at scheduled time intervals. We analyzed the DOX contents in the dialysate by using a Synergy HTX (Bio Tek, Winooski, VT, USA) multifunctional microplate reader.

### 2.9. Cytotoxicity of BGs against HepG2 Cancer Cells

We seeded HepG2 cancer cells and L02 cells at a density of 5000 per well into the wells of a 96-well plate and cultured it in a cell incubator overnight. The next day, we replaced the culture media of the HepG2 cells with fresh DMEM medium (containing 2% FBS) at different pH values (pH 7.4 or 6.5) containing free DOX, BG-DOX, or A-BG-DOX. A similar procedure was applied to the L02 cell line, except that the medium was replaced with RPMI 1640 medium (containing 10% FBS). The concentrations of DOX used were 0, 31.25, 62.5, 125 and 250 ng/mL. After 24 h of incubation, 10 µL of the cell counting kit-8 (Glpbio, Montclair, NJ, USA) reagent was added to each well of the 96-well plate and incubated for 1 h in a cell incubator. Finally, we recorded the absorbance of each well of the plate at 450 nm.

### 2.10. Hemolysis Assay of A-BG-DOX

A hemolytic assay was used to determine the hemocompatibility of A-BG-DOX on mouse red blood cells. First, a fresh blood sample was centrifuged at 1000 rpm for 15 min to obtain red blood cells, then washed several times with PBS, and finally diluted in PBS (1:25). A 150 μL sample of this was then mixed with 150 μL of A-BG-DOX solution (at different concentrations of 20, 10, 5, 2.5, 1.25 μg/mL) in a 1.5 mL plastic centrifuge tube. As a positive control, 0.1% Triton X-100, which lyses almost all red blood cells, was used in this test. PBS was used as a negative control as well. The mixture was incubated at 37 °C for 1 h before being regained and centrifuged at 1000 rpm for 5 min. The absorbance of each sample was determined using a microplate reader and a photometric analysis of supernatant at 540 nm to record hemoglobin release, which represents blood cell damage.

### 2.11. In Vitro Cellular Uptake of A-BG-DOX Analysis by Confocal Fluorescence Microscopy

We used a confocal laser scanning microscope to verify the uptake of A-BG-DOX and free DOX by HepG2 cells. Briefly, the cells were seeded on a confocal dish at a density of 1 × 10^5^ cells and incubated overnight. The next day, we replaced the culture media with fresh media (pH 6.5 and 7.4). Next, A-BG-DOX and free DOX were added to the cells and incubated for 24 h. After that, the cells were washed with PBS and fixed with immunol staining fix solution (Beyotime, Shanghai, China) for 10 min. We added immunostaining permeabilization solution with saponin for 10 min (Beyotime, China). After fixing, permeating, and washing, we incubated the samples with monoclonal ANTI-FLAG M2 antibody (Sigma, USA) diluted at a ratio of 1:3000 overnight at 4 °C, and then the cells were washed three times to remove the excess antibodies. Next, we incubated the cells with goat anti-mouse IgG H&L (Alexa Fluor 647) antibody at a dilution of 1:500. Finally, the samples were stained with Dil (Beyotime, China) and DAPI (Sigma, USA) and studied under a Leica TCS SP8 (Leica, Germany) microscope.

### 2.12. Flow Cytometry Analysis of Cell Apoptosis

After seeding 1 × 10^5^ HepG2 cells/mL onto a 12-well plate and overnight incubation, we replaced the media with fresh media (pH 6.5) containing PBS, 0.5 µg/mL free DOX or A-BG-DOX. After another 8 h of incubation, we harvested the cells and analyzed them by flow cytometry with an Annexin V-FITC apoptosis detection kit (Beyotime, China). Briefly, we gently resuspended the cells in a solution containing 195 µL of Annexin V-FITC binding buffer and 5 µL Annexin V-FITC; we added 10 µL of propidium iodide (PI) working solution to each sample and incubated the cells at room temperature for 10–20 min in the dark. The samples (10,000 events each time) were analyzed by flow cytometry (BD Accuri C6, Franklin Lakes, NJ, USA).

### 2.13. Statistical Analysis

All statistical analyses were performed with GraphPad Prism 8.0 software. The obtained data were expressed in the format of mean ± standard deviation.

## 3. Results and Discussion

### 3.1. Construction of the E. coli Bacterial Ghosts Using Protein E

**Figure 1:**
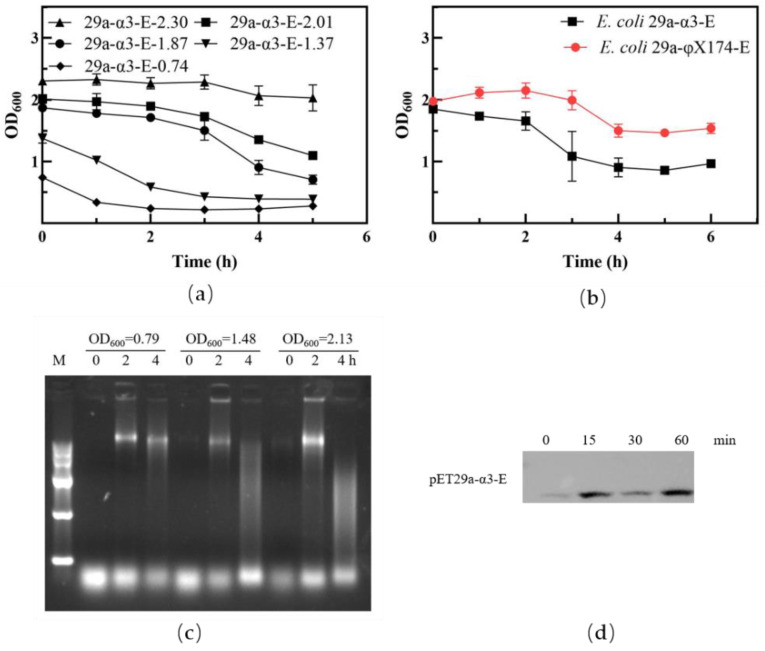
Effect of lysis protein E in bacteria. (**a**) Lysis curve of *E. coli* BL21(DE3) containing the pET29a-α3-E plasmid at different induction starting points. (**b**) Comparative analysis of lysis function in *E. coli* BL21(DE3) containing different lysis plasmids. (**c**) Electrophoretic analysis of genetic material in the supernatant of *E. coli* BL21(DE3) cultures before and after lysis. (**d**) Western blot of lysis protein E from phage α3.

Firstly, we found the lysis system from phage α3 was simple and stable and capable of producing a high yield of *E.* *coli* BGs for an extended period of time. According to our lysis kinetics results (Figure 1a), the protein E from phage α3 was very active in *E. coli* BL21(DE3). The bacteria were being lysed steadily for 3 h after IPTG induction when the culture OD_600_ had reached 0.739, and after that the OD_600_ increased slightly. This may be due to the rapid growth of bacteria at this time, which surpasses the lysis effect of protein E. At OD_600_ culture values between 0.8 and 2.0, the lysis protein E from bacteriophage α3 was active after expression induction. However, inducing the bacteria at OD_600_ values higher than 2.3 led to no decreases in the OD_600_ value. This is probably caused by the increased numbers of dead bacteria and empty BGs in the system.

Figure 1b shows lysis results of the protein E from phage α3 and phage φX174. When the OD_600_ closed to 2.0, the protein E from phage α3 showed stronger lysis activity compared with the protein E from phage φX174, which means the lysis system of protein E from phage α3 is more suitable for producing a high yield of BGs than protein E from phage φX174. In this study, *E. coli* ghosts were produced with high initial cell density, which causes more bacterial genetic materials to be expelled. The agar electrophoretic analysis showed little evidence of bacterial genomes before the lysis, but gene bands appeared 2 h after IPTG induction. The higher the OD_600_ value, the more obvious the bands were. After 4 h of induction, the genomic bands became fuzzy, and we speculate that this was caused by the release of nucleic enzymes that degraded the genomic DNA in the system. (Figure 1c). The lysis protein E of phage α3 is successfully expressed in *E. coli*. Figure 1d shows some leaky expression of lysis protein E before the induction and a strong lysis protein E expression after 15 min of the IPTG induction. However, as the lysis protein is toxic to bacteria, the microorganisms need to regulate its expression, so the expression level of the lysis protein has little difference with the increase of induction time.

Thus, the new protein E from phage α3 is useful in a high density fermentation method to prepare BGs, and it also provides a solid foundation for the application of other lysis proteins to find large-scale preparation methods for BGs.

### 3.2. ATRAM Protein Surface Expression on an Lpp-OmpA Anchor in EcN

**Figure 2:**
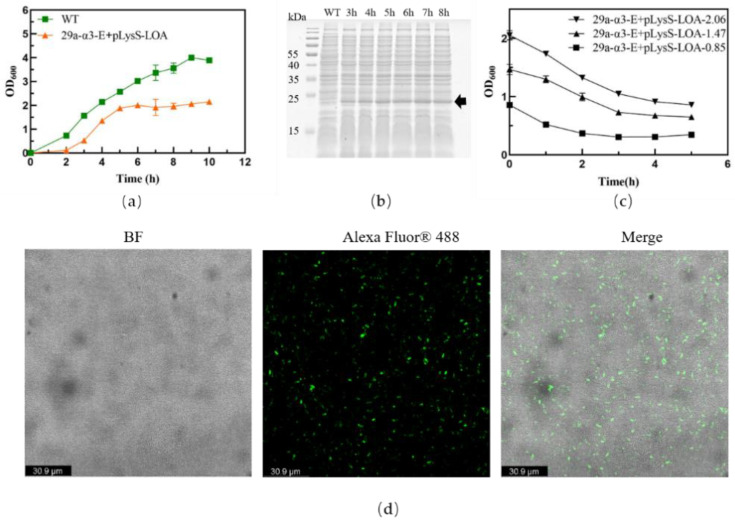
Lpp-OmpA-ATRAM on the EcN/Δ*tnaA*::T7 RNAP membrane surface. (**a**) The growth curves of EcN/Δ*tnaA*::T7 RNAP/pLysS-LOA and EcN/Δ*tnaA*::T7 RNAP (WT). (**b**) SDS-PAGE gel photo showing expression levels of Lpp-OmpA-ATRAM over time. The black arrow indicates the target protein. (**c**) Lysis curves of EcN/Δ*tnaA*::T7 RNAP/ pET29a-α3-E after different induction starting points. (**d**) Confocal images of Lpp-OmpA-ATRAM on the engineered bacterial membrane surfaces. The green fluorescence is derived from the expression of Lpp-OmpA-ATRAM, scar bar is 30.9 μm.

To assess the effect of protein expression on bacterial growth, we detected the expression and location of Lpp-OmpA protein in EcN/Δ*tnaA*::T7 RNAP bacteria. As shown in Figure 2a,b, the most striking characteristic of the EcN/Δ*tnaA*::T7 RNAP/pLysS-LOA bacteria was the plateauing of their early growth velocity after 5 h; in contrast, the EcN/Δ*tnaA*::T7 RNAP bacteria maintained stable growth beyond that time. Moreover, the Lpp-OmpA-ATRAM (~24.0 kDa) protein expression levels remained similar at different time points. It has been reported that the pLysS vector was used to transform *E. coli* BL21 and express the T7 lysozyme, an essential step for the expression of toxic proteins [35]. The low expression level of T7 lysozyme afforded by pLysS has little effect on cell growth, but the level of expression of Lpp-OmpA-ATRAM provided by pLysS increased the growing burden of the EcN/Δ*tnaA*::T7 RNAP bacteria, and probably led to the slowing down of their growth rate. We also performed immunofluorescence assays to further demonstrate the presence of Lpp-OmpA-ATRAM on the membrane surface of the EcN/Δ*tnaA*::T7 RNAP bacteria. After incubation with goat anti-mouse IgG H&L (Alexa Fluor 488), we observed strong green fluorescence signals on the surface of the EcN/Δ*tnaA*::T7 RNAP/pLysS-Lpp-OmpA-ATRAM bacteria (Figure 2d). In short, the Lpp-OmpA-ATRAM can be expressed and is specifically located in the EcN/Δ*tnaA*::T7 RNAP membrane surface.

Next, we verified that the lysis protein E had the expected effects on the transformed bacteria, even in the presence of Lpp-OmpA-ATRAM. At a culture density of OD_600_ = 2.1, the bacteria were effectively lysed and the OD_600_ values decreased significantly (Figure 2d). Therefore, we harvested the BGs after reaching a culture density of OD_600_ = 2.1 to yield BGs with the highest Lpp-OmpA-ATRAM expression on their cell membranes.

### 3.3. Morphology Assessment of EcN Ghosts Expressing Lpp-OmpA-ATRAM

**Figure 3:**
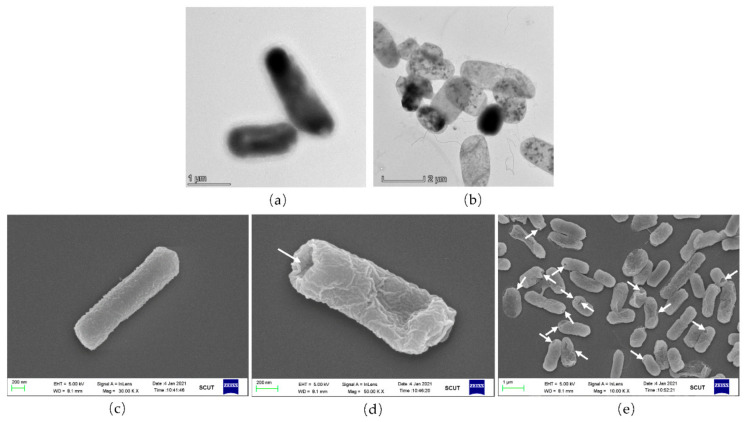
Morphology of A-BGs by TEM and SEM. (**a**) TEM image of the control EcN/Δ*tnaA*::T7 RNAP/pLysS-LOA bacteria. (**b**) TEM image of A-BGs after IPTG induction. (**c**) SEM image of control EcN/Δ*tnaA*::T7 RNAP/pLysS-LOA bacteria. (**d**,**e**) SEM images of a single A-BG and multiple ghosts at lower resolution. The white arrows show the transmembrane tunnels in the bacteria.

To verify the integrity of the EcN ghosts expressing Lpp-OmpA-ATRAM ghosts, a morphology assessment was performed by TEM and SEM. In Figure 3a,b, the undamaged EcN/Δ*tnaA*::T7 RNAP/pLysS-LOA cells are dark because they are unable to transfer light. The EcN/Δ*tnaA*::T7 RNAP/pLysS-LOA cells exhibit semi-transparency on TEM images when they evacuate the majority of their cytoplasmic contents. The SEM image of the EcN/Δ*tnaA*::T7 RNAP/pLysS-LOA bacteria clearly shows trans-membrane tunnels in the ghost cells. Most transmembrane channels are distributed in the bacteria’s poles, but in previous research, lysis tunnels were found at the pore or division zone [33]. Thus, one of the most noticeable features of the produced ghost cells in this investigation was their structural integrity. A wide field of view showed that most of the bacterial cells in this group were lysed and some of them were corrugated because of their lack of cytoplasm (Figure 3c–e). To date, EcN ghosts expressing Lpp-OmpA-ATRAM have been successfully prepared on a large scale with the plasmid pET29a-α3-E.

### 3.4. DOX Packaging and Release Assays

**Figure 4:**
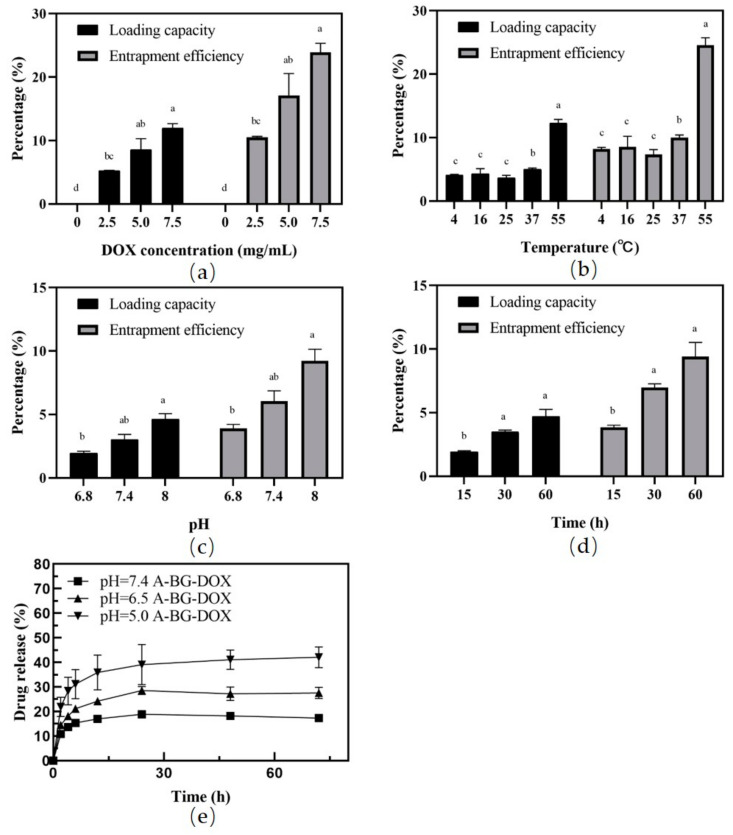
DOX packaging and release assays. Effects of drug concentration (**a**), incubation temperature (**b**), incubation time (**c**), and Tris-HCl buffer pH (**d**) on the drug loading capacity and encapsulation rate. DOX release profiles from A-BG-DOX in PBS at different pH values (**e**). a, b, c, d: significant differences (*p* < 0.05) among different groups.

The loading and leakage of therapeutic agents are key performance features of carriers that should be carefully evaluated (Figure 4). We evaluated the drug-loading capacity and entrapment efficiency of the BGs at different DOX concentrations, lengths, and temperatures of incubation, and at different Tris-HCl buffer pH values. The DOX content in BGs increased with the rising drug concentrations (Figure 4a). Interestingly, the amounts of DOX packaged into the BGs were all similar at lower temperatures. The highest DOX entrapment efficiency (24.6%) and loading capacity (12.28%) were achieved at an incubation temperature of 55 °C (Figure 4b). Temperature affects the fluidity of the bacterial membrane, which promotes DOX molecules binding to the membrane; this accounts for the maximum drug loading at 55 °C (*p* < 0.05). However, high temperatures may negatively affect the morphology of BGs. The membrane releasing behavior in the current study may be accounted for by the BGs obtained by different methods. Moreover, DOX can be loaded into the bacterial ghosts after incubation for 15 min, (Figure 4c). The packaged drug percentage was higher in 0.5 M Tris-HCL buffer (pH 8.0) than that in 0.5 M Tris-HCL buffer (pH 6.8; Figure 4d). We also found that loading of DOX into BGs is drug concentration- and incubation time-dependent within the studied range. This trend is the same as those in previous studies [34].

We measured the release of DOX from A-BGs at pH values 5.0, 6.5, and 7.4 at 0, 2, 4, 6, 12, 24, 48, and 72 h using dialysis and calculating the cumulative release percentage in the dialysate. Figure 4e shows the results of these calculations. We used pH 6.5 and pH 7.4 to simulate the mild-acid microenvironment of tumors and the normal tissue environment, respectively. We found that 10.9% of DOX was released from the A-BG-DOX in two hours at a pH of 7.4; in contrast, 21.9% was released during the same time at pH 5.0. In addition, the drug release rate of BGs was very small at pH 7.4 and stayed below 18.9% after 24 h. However, the drug release rate was significantly higher at pH 5.0 and reached 39.1% after 24 h and 42.1% after 72 h. The leakage rate of DOX from A-BG-DOX indicated that DOX efflux was not occurring to any noticeable extent after packaging into BGs under physiological conditions. When the pH was changed from 7.4 to 5.0, the cumulative drug release of A-BG-DOX increased to a certain extent, and this may have been caused by the increased solubility of DOX in water under those mild-acidic conditions.

### 3.5. Lpp-OMPA-ATRAM Protein Expression on Bacterial Ghosts Enhances Cancer Therapy

**Figure 5:**
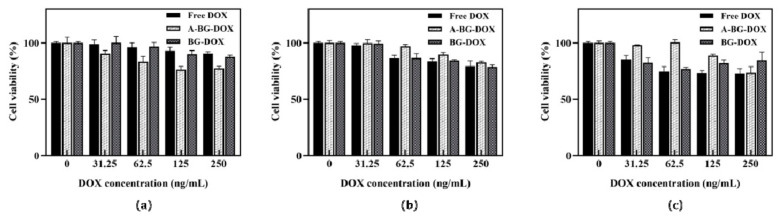
Cytotoxicity of free DOX, BG-DOX, and A-BG-DOX against HepG2 tumor cells in media at (**a**) pH 6.5, (**b**) pH 7.4, and (**c**) L02 cells in normal culture conditions.

Next, to explore the biological application of the vectors, we studied the cytotoxicity of bacterial ghosts bearing various concentrations (0, 31.25, 62.5, 125, 250 ng/mL) of DOX against human hepatoma HepG2 cells and human hepatocyte L02 cells in culture for 24 h. We performed cell viability assays using a cell counting kit-8. At the acidic pH, A-BG-DOX had stronger toxicity against HepG2 cells than free DOX (Figure 5a). The survival rate of cells treated with free DOX (250 ng/mL) was 90.5%, whereas the survival rate of the cells treated with A-BG-DOX decreased to 77.3%, and BG-DOX decreased to 87.5%. Similarly, the cell survival rate after treatment with A-BG-DOX at 31.25 µg/mL was 90.3%, and those after treatment with free DOX and BG-DOX were 98.9% and nearly 100%, respectively. However, as shown in Figure 5b, free DOX showed stronger cytotoxicity against HepG2 cancer cells than A-BG-DOX under physiological conditions. Moreover, we found that HepG2 tumor cells at pH 6.5 had a higher survival rate than those at pH 7.4. This is most likely due to the difficulty of chemotherapy drugs, such as DOX, penetrating membranes and entering cells in an acidic tumor microenvironment, and accumulating outside the cells [36,37]. The A-BG-DOX, as predicted, only showed dose-dependent cytotoxicity on HepG2 cells and no overt cytotoxicity on L02 cells (Figure 5c). On L02 cells, the survival rate of cells treated with free DOX (31.25 ng/mL) was 85.5%, whereas the survival rate of cells treated with A-BG-DOX was 98.0%. These findings show that A-BG-DOX can cause tumor-selective cytotoxicity in cancer cells.

**Figure 6:**
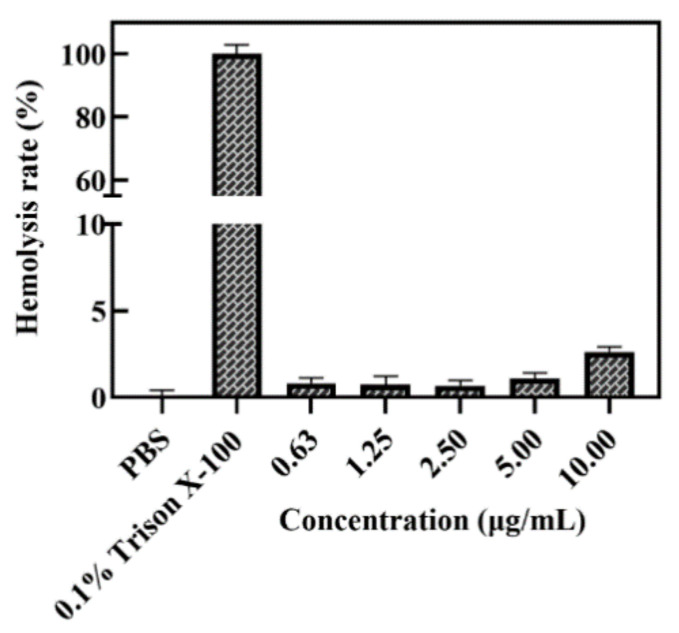
Hemolysis histogram of 0.1% Triton X-100 (positive control), PBS (negative control), and different concentrations of A-BG-DOX.

A homolysis test with red blood cells was performed to characterize the blood compatibility and the possibility of in vivo application of the A-BG-DOX. The results of the tests revealed that there was no hemolytic toxicity of A-BG-DOX at the tested concentration (10 μg/mL), with a hemolysis rate of less than 5%, whereas 0.1% triton X-100 lysed almost all red blood cells (Figure 6). These findings show that A-BG-DOX is extremely safe in blood circulation.

**Figure 7:**
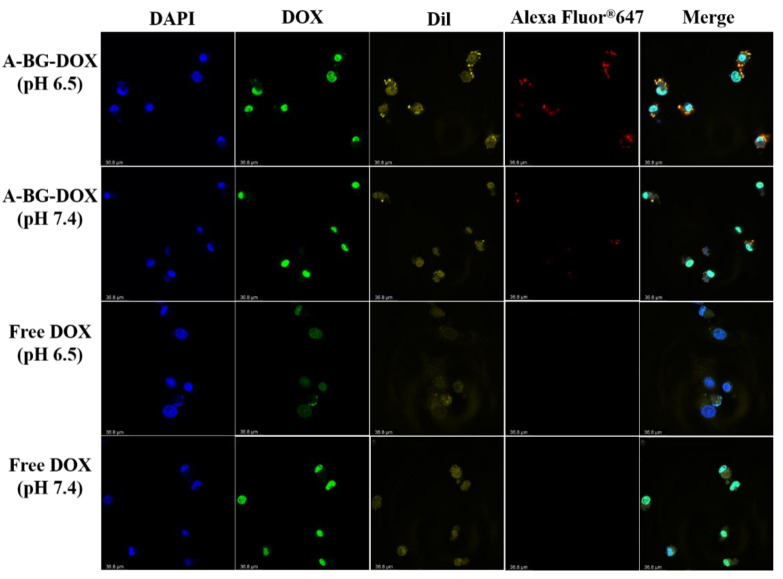
In vitro fluorescence imaging of HepG2 cells after treatment with A-BG-DOX and free DOX in their culture medium at pH 6.5 and 7.4; scar bar is 36.8 μm.

We studied the intracellular localizations of A-BG-DOX and free DOX in HepG2 cells cultured in media at pH 6.5 and 7.4. The cells were treated with A-BG-DOX and free DOX for 24 h and co-stained with goat anti-mouse IgG H&L (Alexa Fluor 647), Dil, and DAPI. As shown in Figure 7, we detected a bright-red fluorescence in cultured HepG2 cells (pH 6.5 and 7.4) pretreated with A-BG-DOX after 24 h incubations, whereas no red fluorescence of free DOX was detected under the same conditions.

The confocal laser images showed strong red fluorescence in HepG2 cells after incubation with A-BG-DOX (pH 6.6 and pH 7.4). It was speculated that the ATRAM peptide can bind to the surface of lipid membranes at pH 7.4, and it inserts itself into the lipid bilayer as a transmembrane α-helix at a moderately acidic pH similar to the extracellular pH in solid tumors [38]. The fluorescence could be also seen inside the cells. EcN is a commonly used probiotic for tumor therapy and it can colonize tumors in rodent models of liver metastasis by transiting the gut epithelium to enter the bloodstream [39]. According to these results, we speculate that A-BG-DOX enters lesion sites in a liver tumor by passive transport and that the presence of ATRAM increases the targeting ability of BGs to acidic tumor environments.

**Figure 8:**
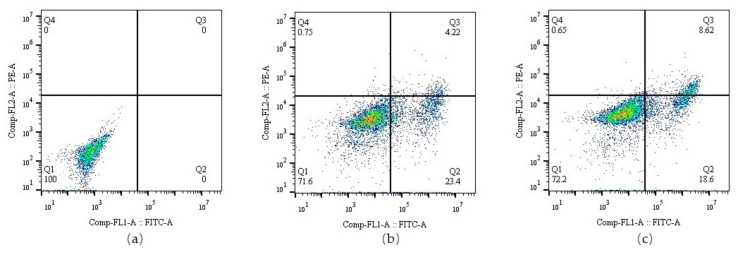
Flow cytometry chart representing the percentage of apoptotic HepG2 tumor cells treated with (**a**) PBS, (**b**)A-BG-DOX (pH 6.5), and (**c**) free DOX (pH 6.5).

To study the percentage of apoptotic and necrotic cells induced by A-BG-DOX at pH 6.5, we performed apoptosis assays on treated HepG2 cells using flow cytometry. The total cells were divided into four populations including normal cells (annexin V−/PI−, Q1), early apoptotic cells (annexin V+/PI−, Q2), late apoptotic cells (annexin V+/PI+, Q3), and necrotic cells (annexin V−/PI+, Q4). Treatment with 0.5 µg/mL of A-BG-DOX left 71.6% of the cells alive, while 23.4% had undergone early apoptosis and 4.97% had undergone late apoptosis/necrosis. Moreover, treatment with free DOX left 72.2% of the cells alive, 18.6% in an early apoptosis phase, and 12.8% in late apoptosis and necrosis phases (Figure 8a–c). In short, the A-BG-DOX treatment resulted in more cells shifting toward early apoptosis than treatment with free DOX (pH 6.5).

## 4. Conclusions

In summary, we present a new bacterium-based therapeutic strategy with BGs that produced high-quality EcN ghosts on a large scale and sequentially expressed the Lpp-OmpA-ATRAM for selective cancer cell therapy. Most importantly, we offer a means for bionic bacteria construction in the future, and the strategy will help develop therapeutics to treat cancer and infectious diseases due to their low-cost, simple production, and gene editing feasibilities.

## Supplementary Materials

The following supporting information can be downloaded at: https://www.mdpi.com/article/10.3390/bioengineering9090433/s1, Table S1: Plasmids from this study and their Addgene accession numbers.
