# Supplementary material for: Construction and In Vitro Evaluation of a Tumor Acidic pH-Targeting Drug Delivery System Based on Escherichia coli Nissle 1917 Bacterial Ghosts
Source: Bioengineering (Basel). 2022 Sep 2;9(9):433. doi: 10.3390/bioengineering9090433 (PMC9495381; doi:10.3390/bioengineering9090433)
Supplement: Supplementary file 1 [file bioengineering-09-00433-s001.zip › bioengineering-1727080-supplementary.pdf]

Supplementary Materials

Construction and In Vitro Evaluation of a Tumor Acidic pH-Targeting Drug Delivery System Based on *Escherichia coli* Nissle 1917 Bacterial Ghosts

Yi Ma [1,2,*], Qiying Liu [1], Aihua Hu [3], Shoujin Jiang [1], Sijia Wang [1], Ran Liu [4], Kun Han [4] and Jufang Wang [1,*]

[1] School of Biology and Biological Engineering, South China University of Technology, Guangzhou 510006, China
[2] Guangdong Provincial Key Laboratory of Fermentation and Enzyme Engineering, South China University of Technology, Guangzhou 510006, China
[3] The Third Affiliated Hospital of Guangzhou Medical University, Guangzhou 510140, China
[4] Jiangsu Key Biotechnology Co., Ltd., Xuzhou 221100, China
* Correspondence: bimayikobe@scut.edu.cn (Y.M.); jufwang@scut.edu.cn (J.W.)

Table S1. Plasmids from this study and their Addgene accession numbers.

Plasmid name	Addgene accession numbers.
pLysS-LOA	187934
pET29a-α3-E	187935
